# Podocyte specific deletion of PKM2 ameliorates LPS-induced podocyte injury through beta-catenin

**DOI:** 10.1186/s12964-022-00884-6

**Published:** 2022-05-30

**Authors:** Mohammed Alquraishi, Samah Chahed, Dina Alani, Dexter L. Puckett, Presley D. Dowker, Katelin Hubbard, Yi Zhao, Ji Yeon Kim, Laurentia Nodit, Huma Fatima, Dallas Donohoe, Brynn Voy, Winyoo Chowanadisai, Ahmed Bettaieb

**Affiliations:** 1 Department of Nutrition, The University of Tennessee Knoxville, 1215 Cumberland Avenue, 229 Jessie Harris Building, Knoxville, TN 37996-0840 USA; 2 Department of Pathology, University of Tennessee Medical Center, Knoxville, TN 37920 USA; 3 Department of Pathology, University of Alabama at Birmingham, Birmingham, AL USA; 4 Tennessee Agricultural Experiment Station, University of Tennessee Institute of Agriculture, Knoxville, TN 37996-0840 USA; 5 Graduate School of Genome Science and Technology, University of Tennessee, Knoxville, TN 37996-0840 USA; 6 Department of Nutrition, Oklahoma State University, Stillwater, OK 74078 USA; 7 Department of Biochemistry, Cellular and Molecular Biology, University of Tennessee, Knoxville, TN 37996-0840 USA; 8 Present Address: Department of Community Health Sciences, King Saud University, Riyadh, Saudi Arabia; 9 Present Address: Kellogg Eye Center, University of Michigan, Ann Arbor, MI 48105 USA

**Keywords:** Proteinuria, Pyruvate kinase M2, Podocyte, β-Catenin

## Abstract

**Background:**

Acute kidney injury (AKI) is associated with a severe decline in kidney function caused by abnormalities within the podocytes' glomerular matrix. Recently, AKI has been linked to alterations in glycolysis and the activity of glycolytic enzymes, including pyruvate kinase M2 (PKM2). However, the contribution of this enzyme to AKI remains largely unexplored.

**Methods:**

Cre-loxP technology was used to examine the effects of PKM2 specific deletion in podocytes on the activation status of key signaling pathways involved in the pathophysiology of AKI by lipopolysaccharides (LPS). In addition, we used lentiviral shRNA to generate murine podocytes deficient in PKM2 and investigated the molecular mechanisms mediating PKM2 actions in vitro.

**Results:**

Specific PKM2 deletion in podocytes ameliorated LPS-induced protein excretion and alleviated LPS-induced alterations in blood urea nitrogen and serum albumin levels. In addition, PKM2 deletion in podocytes alleviated LPS-induced structural and morphological alterations to the tubules and to the brush borders. At the molecular level, PKM2 deficiency in podocytes suppressed LPS-induced inflammation and apoptosis. In vitro, PKM2 knockdown in murine podocytes diminished LPS-induced apoptosis. These effects were concomitant with a reduction in LPS-induced activation of β-catenin and the loss of Wilms’ Tumor 1 (WT1) and nephrin. Notably, the overexpression of a constitutively active mutant of β-catenin abolished the protective effect of PKM2 knockdown. Conversely, PKM2 knockdown cells reconstituted with the phosphotyrosine binding–deficient PKM2 mutant (K433E) recapitulated the effect of PKM2 depletion on LPS-induced apoptosis, β-catenin activation, and reduction in WT1 expression.

**Conclusions:**

Taken together, our data demonstrates that PKM2 plays a key role in podocyte injury and suggests that targetting PKM2 in podocytes could serve as a promising therapeutic strategy for AKI.

***Trial registration*:**

Not applicable.

**Video abstract**

**Supplementary Information:**

The online version contains supplementary material available at 10.1186/s12964-022-00884-6.

## Background

Acute kidney injury (AKI) describes a sudden and severe loss of renal function that can develop over the course of hours or days. Overall, the occurrence of AKI has markedly increased in the US over the past 2 decades, with approximately 4 million people in the US hospitalized for AKI in 2014 [1]. Although treatment typically restores kidney function, AKI increases the possibility of subsequently developing proteinuria, a significant risk factor for chronic kidney disease and cardiovascular events [2]. Accordingly, AKI is associated with an increased likelihood of the need for long-term care, lifetime risk of hospitalization, and elevated health care costs [1].

Damage to podocytes is the critical initiating event linked to the development of proteinuria during AKI [3]. Podocytes have proven to be crucial in the prevention of proteinuria through their role in maintaining the structural integrity of the glomerular filtration membrane. Foot processes of these specialized epithelial cells create the slit diaphragms through which filtrate passes into the proximal tubules [4, 5]. Damage to podocytes compromises the filtration barrier, resulting in protein loss in urine [6]. Podocytes are particularly vulnerable to stressors in the blood due to their anatomical arrangement within the filtration barrier. Pathogens and other stress signals are sensed by podocytes, eliciting the release of inflammatory cytokines and recruitment of immune cells into the glomerulus [7, 8]. If unresolved, inflammation can damage the filtration barrier, resulting in chronic proteinuria and increased risk of renal disease. In addition, toll-like receptors (TLR) that recognize endotoxin lipopolysaccharides (LPS) and other microbial stress signals are expressed in podocytes and distributed throughout the nephron. Activation of these receptors by pathogens in the renal filtrate elicits local release of inflammatory cytokines, microvascular alterations, and metabolic disruptions that further impede renal function [9].

Emerging studies indicate that LPS rapidly induces aerobic glycolysis in target cells, and that this metabolic shift is necessary for the inflammatory response. Colorectal cells, macrophages, dendritic cells, and other immune cells rapidly switch from mitochondrial-derived production of ATP to aerobic glycolysis when exposed to LPS [10–15]. Inhibiting this Warburg-like metabolic shift suppresses the subsequent release of inflammatory cytokines, demonstrating a causal connection between glycolysis and inflammation during the acute response to LPS. Similar relationships have been described in an in vivo LPS-induced model of lung injury. Injecting mice with 2-deoxyglucose, an inhibitor of glycolysis, prior to the administration of LPS, attenuated infiltration of neutrophils, suppressed the expression of inflammatory markers, and limited pathological injury in the lung [16]. Experimental sepsis has also been shown to induce a shift toward aerobic glycolysis in the kidney. Administering LPS to mice prompted a significant rise in glycolytic metabolism in the renal cortex that preceded the decline in renal function [17]. This shift in metabolism occurred within three hours of LPS treatment, consistent with rapid induction observed in other cells and tissues. In the kidneys from a murine cecal puncture model of sepsis, metabolome profiling revealed a significant increase in glycolytic metabolites with a corresponding reduction in TCA cycle intermediates [18] Based on these studies, and on findings in other cell types and tissues, Gomez et al. proposed a model in which renal cells undergo metabolic reprogramming as part of an early pro-inflammatory phase, and furthermore, that this response is linked to mitochondrial injury that occurs in the kidney during the early stages of sepsis [19].

The glycolytic enzyme pyruvate kinase M2 (PKM2) has emerged as a key enzyme that connects glycolysis to inflammation during sepsis. PKM2 is one of four isoforms (L, R, M1, and M2) of the enzyme pyruvate kinase, which catalyzes the final and rate-limiting step in glycolysis. LPS induces the expression of PKM2 in macrophages and other cell types and inhibiting this induction through either pharmacological or genetic approaches prevents initiation of the inflammatory cascade [11, 12, 20, 21]. Both, genetic deletion of PKM2 in myeloid cells and inhibition using shikonin in mice, protect from endotoxic shock and septic death, demonstrating a critical role for this enzyme in sepsis [22, 23]. The inflammatory role of PKM2 may include both its classical glycolytic function and its non-metabolic actions [24]. Unlike other isoforms, the kinase activity of PKM2 extends beyond glycolysis, including phosphorylation of proteins essential to the inflammatory response [25]. In colorectal cancer cells, for example, phosphorylation of STAT3, rather than phosphoenolpyruvate, by PKM2 mediates the inflammatory response to LPS [14]. PKM2 can also regulate inflammation through transcriptional control of cytokine release. In macrophages, LPS induces the formation of a PKM2 and Hypoxia-inducible factor 1-alpha (HIF-1α) complex, forming a dimer capable of translocating to the nucleus to regulate the transcription of *Tnf-a*, *IL-1b*, and other cytokine genes [26, 27]. Notably, Wu and colleagues demonstrated that PKM2 inhibition using shikonin ameliorated LPS-induced AKI through downregulating HIF-1α expression and inhibiting apoptosis in tubular epithelial cells [28]. It is noteworthy to mention that, according to previously published studies, PKM2 is the main pyruvate kinase isoform expressed in podocytes [29]. As such, PKM2 represents an emerging link between metabolism and inflammation during sepsis. Additionally, while podocytes play a critical role in the pathogenesis of AKI, the influence of LPS on podocyte metabolism and the contribution of PKM2 to sepsis-induced AKI are not known. In the current study, we investigated the role of PKM2 in podocyte homeostasis in response to LPS and delineated the potential molecular mechanisms.

## Methods

### Reagents

Unless indicated otherwise, we obtained most chemicals from Sigma‐Aldrich (St. Louis, MO, USA). RPMI164 medium, penicillin–streptomycin, fetal bovine serum (FBS), and trypsin were obtained from Invitrogen (Carlsbad, CA, USA). Hygromycin was purchased from Research Products International Corp. (RPI Corp.; Mount Prospect, IL). Primary and secondary antibodies with their dilutions, hosts, and sources are summarized in Table 1. Forward and reverse primers used for quantitative real-time polymerase chain reaction (qRT-PCR) are listed in Table 2 and were purchased from Fisher Scientific (Hampton, NH).

**Table 1:** List of antibodies used in the reported experiments:

Antibodies	Source	Catalog number	MW (kDa)	Host	Dilution
β-catenin	Santa Cruz Biotechnology	sc-7963	90	Mouse	1:1000
Phospho-β-catenin ^S33^	Santa Cruz Biotechnology	sc-57535	90	Mouse	1:1000
Phospho-β-catenin ^Y333^	AbCam	Ab119363	90	Rabbit	1:1000
CHOP	Santa Cruz Biotechnology	sc-7351	31	Mouse	1:5000
Cleaved Caspase-3	Cell Signaling Technology	9662	17	Rabbit	1:5000
Cleaved Caspase-12	AbCam	Ab62484	43	Rabbit	1:1000
Cleaved Caspase-7	Santa Cruz Biotechnology	sc-56063	20	Mouse	1:1000
c-Myc	Santa Cruz Biotechnology	Sc-40	67	Mouse	1:1000
IKKα	Cell Signaling Technology	2682	87	Rabbit	1:1000
IκBα	Cell Signaling Technology	4814	40	Mouse	1:1000
NF-κBp65	Cell Signaling Technology	8242	65	Rabbit	1:1000
Nephrin	Santa Cruz Biotechnology	sc-376522	170	Mouse	1:500
WT1	Santa Cruz Biotechnology	sc-7385	52	Mouse	1:1000
JNK1/2	Santa Cruz Biotechnology	sc-7345	46/54	Mouse	1:1000
p38	Santa Cruz Biotechnology	sc-7972	42	Mouse	1:1000
PKM2	Cell Signaling Technology	4053	61	Rabbit	1:5000
PKM1	Millipore-Sigma	SAB4200094	61	Rabbit	1:1000
PKM1/2	AbCam	Ab181693	61	Goat	1:1000
Phosphp-IKKα^S176/180^	Cell Signaling Technology	2697	87	Rabbit	1:1000
Phosoho-IκBα^S32^	Cell Signaling Technology	2852	40	Rabbit	1:1000
pNF-κBp65^S36^	Cell Signaling Technology	3033	65	Rabbit	1:1000
Phospho-JNK1/2^T183/Y185^	Santa Cruz Biotechnology	sc-6254	46/54	Mouse	1:1000
Phospho-P38^T180/Y182^	Cell Signaling Technology	4511	43	Mouse	1:10,000
Phospho-PKM2^S37^	ThermoFisher	PA5-37684	61	Rabbit	1:500
Phospho-PKM2^Y105^	Cell Signaling Technology	3827	61	Rabbit	1:1000
β-Actin	Santa Cruz Biotechnology	sc-47778	44	Mouse	1:20,000

**Table 2:** List of primers used in qRT-PCR experiments

Gene	Forward 5′–> 3′	Reverse 5′–> 3′
Pkm (PKM2 isoform)	CACTTGCAGCTATTCGAGGAA	CGAGTCACGGCAATGATAGGA
Pkm (PKM1 isoform)	CATGTTCCACCGTCTGCTGTT	CGAGTCACGGCAATGATAGGA
Tbp	TTGGCTAGGTTTCTGCGGTC	GCCCTGAGCATAAGGTGGAA

### Cell culture

E11 murine podocyte cells were obtained from Cell Lines Service (Eppelheim, Germany) and cultured at 33ºC in a humidified atmosphere of 10% CO_2_. Cells were grown in RPMI164 medium supplemented with 10% fetal bovine serum, Glutamax (2 mM), and sodium pyruvate (1 mM). Differentiation of podocytes was induced as previously described with modification [30]. Briefly, cells were transferred to 37 °C and cultured for an additional period of 15 days. By then, cells exhibit a fully differentiated phenotype as judged by the expression of podocyte markers, namely nephrin and podocin. Cell culture media was replaced every 48 h and all experiments on cells were conducted at least three times (between passages 3 and 8). PKM2 silencing in E11 cells was achieved using three different hairpins (GeneCopoeia, Inc.; Rockville, MD). Lentivirus packaging system (GeneCopoeia) was used to generate lentiviruses in HEK293FT cells (GeneCopoeia) following the manufacturer’s guidelines, and then used to infect E11 podocytes. Cells deficient in PKM2 were selected using hygromycin (200 μg/ml). Cells with PKM2 knockdown (KD) were reconstituted using lentivirus particles overexpressing a human wild type PKM2, selected using geneticin (G418; 400 µg/ml) for three weeks, and used as controls (M2R). Unless indicated otherwise, cells were treated with LPS (5 ug/ml; Sigma-Aldrich) for 24 h.

### Mouse studies

All mice strains (*pkm*2^fl/fl^, podocin-Cre, and C57Bl/6J) were obtained from the Jackson Laboratory, sustained on a 12 h light–dark cycle, and fed ad libitum a standard lab chow diet (Purina lab chow, # 5001). To generate mice with specific deletion of PKM2 in podocytes, we crossed *pkm*2^fl/fl^ to mice expressing Cre under the promoter of *podocin*. Genotyping for the presence of Cre and pkm2 floxed allele was conducted on DNA isolated from tails, using polymerase chain reaction (PCR). For LPS-induced glomerular injury, we injected 8–12 week old PKM2 knockout (*pkm*2^fl/fl^ Pod-cre + ; KO) and control (*pkm*2^fl/fl^; Crtl) age matched male mice with a single dose of LPS intraperitoneally [16 mg/kg of body weight dissolved in 0.9% sterile phosphate buffered saline (PBS)]. 22 h after injection, urine samples were collected into 50 ml conical tubes. The urine samples were centrifuged for 1 min at 3000 rpm, then stored at − 80 °C until further analyses. Mice were sacrificed 2 h after urine collection, and kidneys were harvested and used for biochemical and histological experiments. All mice studies were conducted in accordance with federal regulations upon approval from the Institutional Animal Care and Use Committee at the University of Tennessee, Knoxville.

### Metabolic analysis

Blood samples were collected at the time of sacrifice and evaluated for blood urea nitrogen (BUN) using the Urea Nitrogen Colorimetric Detection Kit from Arbor Assays (Ann Arbor, MI). Additionally, the BCG Albumin and Creatinine Assay Kits (Sigma-Aldrich) were used to measure serum and urine albumins, as well as creatinine levels, respectively, according to the manufacturer's recommendations.

### Podocyte isolation and cell culture

We isolated primary podocytes from C57BL6/J control wild-type mice, and mice lacking PKM2 in podocytes as previously described [31, 32]. Briefly, four kidneys from four different animals were harvested, decapsulated, and minced in Krebs–Henseleit saline (KHS) buffer containing 119 mM NaCl, 4.7 mM KCl, 1.9 mM CaCl_2_, 1.2 mM KH_2_PO_4_, 1.2 mM MgSO_4_·7H_2_O, and 25 mM NaHCO_3_, pH: 7.4. Samples were then centrifuged for 10 min at 2000 rpm, washed twice using the KHS buffer, passed through a 250 μm sieve, and then pelleted for 10 min at 2000 rpm. Next, samples were digested in a Hanks’ balanced salt solution (HBSS) containing collagenase D (0.1%), trypsin (0.25%), and DNase I (0.01%) for 30 min in a 37 °C shaking water bath. The digested samples were filtered through a 100 μm sieve placed on the top of a 53 μm sieve. Finally, samples were centrifuged for 5 min at 4000 rpm in 4 °C, and the pelleted cells were either flash-frozen in liquid nitrogen or lysed in radioimmunoprecipitation assay (RIPA) buffer for biochemical analyses.

### Metabolic flux analyses

Extracellular Acidification Rate (ECAR), a proxy for glycolysis, was measured using Seahorse XF24 extracellular analyzer (Seahorse Bioscience, MA) as previously described [33]. Fully differentiated E11 cells were seeded at identical numbers and cultured in XF24 plates for 24 h. Six hours prior to the assay, cells were treated with LPS and then transferred to a non-CO_2_ incubator (37 °C) for one additional hour before the Seahorse assay. All Seahorse experiments were performed with identical conditions. In brief, glucose at 5 mM final concentration was injected and the change in ECAR was measured from baseline. Then, oligomycin was injected and ECAR was measured again. Finally, 2-deoxyglucose was injected to block glycolysis. The ECAR was normalized to total protein in each well.

### Kidney injury scoring

Freshly harvested kidneys were fixed in 4% paraformaldehyde for 24 h at 4 °C. The fixed tissue was processed and paraffin embedded. Then 4–5 μm thick sections were cut, deparaffinized in xylene, and stained with Periodic Acid–Schiff reagent (PAS) (Sigma) according to the manufacturer's instructions. Each section was examined at high power (40X) for 10 different fields in the renal cortex. Morphological alterations and the magnitude of acute tubular injury were assessed following an arbitrary scale as previously described [34, 35]. Briefly, a score of 0 indicates a normal tubule; a score of 1 indicates tubular lumen dilatation and loss of brush border; a score of 2 indicates tubular epithelial cells vacuolization; a score of 3 indicates apical blebbing of tubular epithelial cells; and a score of 4 indicates tubular epithelial cells sloughing. Finally, the extent of acute tubular injury in each histologic section was calculated as follows: number of tubules with a score higher than 0 X corresponding score/total numbers of tubules examined, and then expressed as percentages.

### Immunofluorescence

For immunofluorescence microscopy, freshly harvested kidney tissues from PBS and LPS-treated WT and PKM2 KO mice were fixed in 10% formalin and embedded in paraffin. 5 μM sections were deparaffinized by three washes in freshly prepared 100% xylene (3 min each) followed by three washes in freshly prepared decreasing concentrations of ethanol (95, 90, 75, and 50%; 3 min each wash). Sections were then stained with anti-PKM2 or anti-nephrin antibodies, and the blue-fluorescent DNA stain; 4', 6-Diamidino-2-Phenylindole, Dihydrochloride (DAPI). Detection was conducted using the appropriate fluorescein-conjugated secondary antibodies and visualized by the Leica DMI8 inverted fluorescent microscope.

### Protein extraction and immunoblots

Podocytes and kidney samples were lysed in RIPA buffer containing freshly prepared solutions of protease and phosphatase inhibitors. Next, samples were sonicated twice (10 s each) on ice and cleared by 10 min centrifugation at 12,500 rpm at 4 °C. Total protein concentration was determined using the bicinchoninic acid (BCA) assay kit (Pierce Chemical, Dallas, TX, USA). A total of 10–50 μg of proteins were resolved in electrophoresis then transferred to polyvinylidene fluoride (PVDF) membranes. Membranes were blocked with TBST containing 5% BSA and 0.1% tween, pH: 7.4 for 45–60 min at room temperature (RT) before incubation with the indicated primary antibodies (Table 1) for 1 h at RT. After three wash cycles with TBST (5 min each), membranes were incubated with the appropriate secondary antibodies for 1 h at RT, then washed 4 times (15 min each) with TBST. Luminata™ Western Chemiluminescent HRP Substrate (Millipore Corp., Billerica, MA, USA) was used to visualize proteins and the band's intensity was quantified using Fluorchem software (Alpha Innotech Corp., San Jose, CA, USA).

### RNA isolation and quantitative real-time PCR

mRNA was extracted from kidney and cells using TRIzol reagent (Invitrogen), pelleted in RNase-free water following the manufacturer's instructions, and quantified using NanoDrop® ND1000 spectrophotometer (Thermo Fisher Scientific Inc., Piscataway, NJ). 5 μg of RNA was used for cDNA synthesis using iScript™ cDNA Synthesis Kit (BioRad; Hercules, CA). All gene expression analysis was performed by qRT-PCR using the SsoAdvanced™ Universal SYBR® Green Supermix (BioRad) and BioRad CFX96™ system, as previously described [31]. We evaluated the relative abundance of mRNA of the indicated genes using respective primers (Table 2) via the ∆∆^CT^ method. For each gene, the expression of the corresponding mRNA was normalized to the *tata-box binding protein (tbp)* as previously described [36].

### Morphological analysis of apoptosis

Changes in chromatin condensation in cells upon LPS exposure were assessed as previously described [37] with modification. Briefly, cells were exposed to LPS for the indicated duration then washed with PBS and labeled with Hoechst 33258 (50 µg/ml in PBS). Condensed nuclear chromatin was visualized using fluorescence microscopy (Leica DMI8, Leica Microsystems Inc. Buffalo Grove, IL).

### Caspase 3 activity

Following LPS treatment, caspase-3 activity was determined as previously described with modification [38]. Briefly, 5 × 10^6^ cells were lysed using the repeated freeze–thaw cycles method in 50 μl cell lysis buffer (50 mM HEPES, pH 7.4, 100 mM NaCl, 0.1% CHAPS, 1 mM DTT, 0.1 mM EDTA) in a 96 well plate. During each cycle, cells were incubated at -20 °C for 30 min. Next, 50 μl of the caspases assay buffer solution (50 mM HEPES, pH 7.4, 100 mM NaCl, 0.1% CHAPS, 10 mM DTT, 0.1 mM EDTA and 10% glycerol) containing 50 µM of the caspase-3 substrate (Ac-DEVD-pNA; Calbiochem, La Jolla, CA) was added to each well and incubated at 37 °C for 30 min. Caspase-3 activity was determined by measuring the absorbance at 405 nm using the Synergy™ HTX Multi-Mode microplate reader and was normalized to control cells not exposed to LPS.

### Statistical analyses

Statistical analyses were conducted using JMP data analysis software (SAS Institute Inc., Cary, NC, USA), and an unpaired heteroscedastic two‐tailed Student's t test was used for two groups comparisons, while ANOVA (with Tukey–Kramer post hoc analysis) was used for multi-groups comparison. Data are shown as means + standard error of the mean (SEM). The level of significance was set at *p* ≤ 0.05, while *p* ≤ 0.01 was set for highly significant data. Single symbol (*) refers to *p* ≤ 0.05, and double symbols (**) refer to *p* ≤ 0.01.

## Results

### Changes in PKM2 expression and phosphorylation in response to LPS treatment

**Fig. 1:**
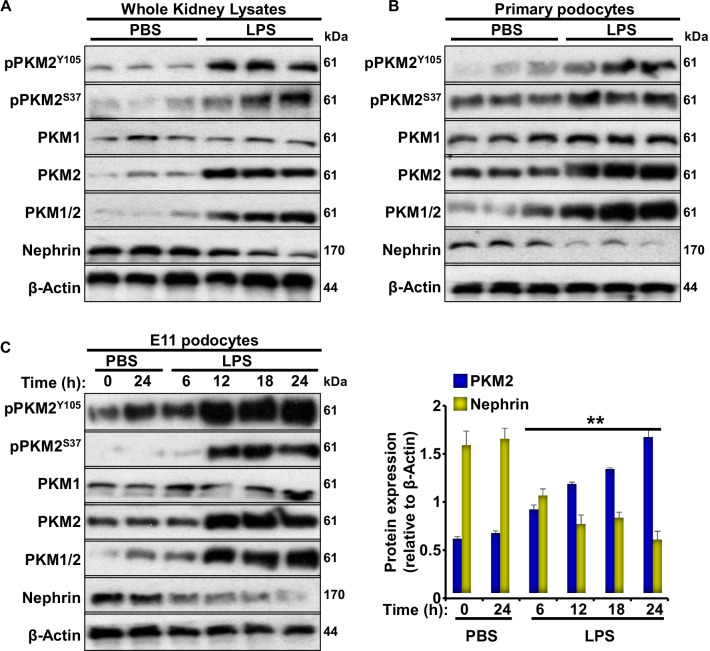
LPS increases PKM2 phosphorylation and expression levels in renal tissue and cultured podocytes. Representative immunoblots of pPKM2^Y105^, pPKM2^S37^, PKM2, PKM1, PKM1/2, nephrin, and β-Actin as a loading control in **A** total kidney lysates harvested from C57bl6/J wild type mice 24 h after PBS or LPS injection (n = 6/group) and **B** primary podocytes isolated from C57bl6/J wild type mice 24 h after PBS or LPS injection (n = 12; 4 animals/lane). **C** Representative immunoblots (left panel) of pPKM2^Y105^, pPKM2^S37^, PKM2, PKM1, PKM1/2, nephrin, and β-Actin in cultured murine E11 podocytes in response to PBS or LPS treatment for the indicated duration. Bar graphs (right panel) displays changes in PKM2 and nephrin levels normalized to β-Actin from three independent experiments. *p < 0.05, **p < 0.01 indicate a significant difference between cells treated with LPS and non-treated cells

Recent studies have proposed that podocytes may exhibit a phasic switch from oxidative phosphorylation (OXPHOS) to aerobic glycolysis similar to Warburg metabolism under non-physiological conditions such as the exposure to high glucose concentrations [39]. Thus, we examined changes in PKM2 expression in total kidneys lysates and primary podocytes isolated from WT mice in response to LPS. Exposure to LPS resulted in a significant increase in renal PKM2 protein levels (Fig. 1A). Additionally, and in agreement with previous reports [31, 40], LPS decreased nephrin levels in whole kidney lysates and primary podocytes compared to control (Ctrl) mice injected with PBS. Consistent with these findings, renal and primary podocyte PKM2 mRNA were significantly higher in LPS-treated mice compared to controls (Additional file 1: Figure S1A). Moreover, E11 podocytes exposed to LPS exhibited a significant increase in glycolysis compared to control cells (Additional file 1: Figure S1B–C).

Based on previous reports, the increase in PKM2 phosphorylation at tyrosine-105 and serine-37 is indicative of decreased PKM2 enzymatic activity and enhanced nuclear accumulation [41, 42]. Here, we show that increased PKM2 expression was paralleled with increased phosphorylation at both tyrosine and serine sites. These results suggest that both enzymatic and nuclear functions of PKM2 were altered in response to LPS. Consistent with this observation, PKM2 expression and phosphorylation increased after LPS treatment in primary podocytes isolated from C57Bl6 control mice (Fig. 1B). To examine whether the use of cell-based systems recapitulates our in vivo and ex vivo findings, we examined the expression and phosphorylation of PKM2 in E11 murine podocytes cell line exposed to LPS for different time points (6, 12, 18, and 24 h). Consistent with the in vivo findings, immunoblotting revealed a significant time-dependent induction of PKM2 expression and phosphorylation in response to LPS, paralleled with a reduction in nephrin protein levels (Fig. 1C). These data demonstrate the regulation of PKM2 expression and phosphorylation in response to LPS and suggest that modulation of PKM2 expression and/or activity might be relevant to acute podocyte injury.

### Specific deletion of PKM2 in podocytes prevents LPS-induced alterations in renal function

**Fig. 2:**
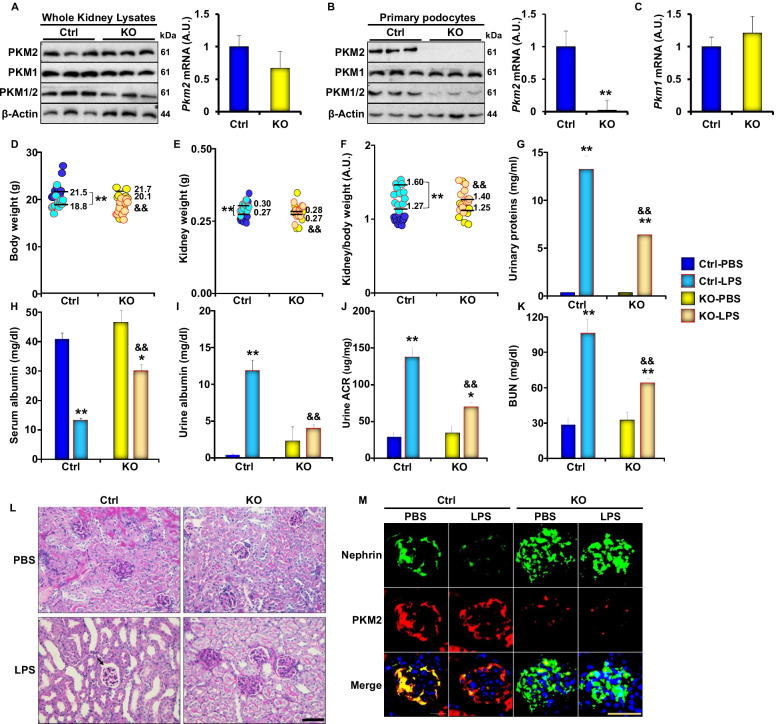
PKM2 podocyte deletion ameliorates LPS induced proteinuria and kidney injury. **A** Representative immunoblots (left panel) of PKM2, PKM1, PKM1/2, and β-Actin in whole kidney lysates from wild type (Ctrl) and podocyte PKM2 knockout mice (KO). mRNA levels (right panel) of *Pkm2* in total kidney lysates of Ctrl and KO mice (n = 6 group). **B** Representative immunoblots (left panel) of PKM2, PKM1, PKM1/2, and β-Actin in primary podocytes isolated from Ctrl and KO mice. The right panel is the mRNA level of *Pkm2 and Pkm1* **C** in primary podocytes (n = 12; 4 animals/lane). **D** Body weights, **E** kidney weights, and **F** kidney to body weight ratio of wild type (Ctrl) and podocyte PKM2 knockout mice (KO) mice, under PBS and LPS treated states. Assessment of **G** total urinary proteins levels, **H** serum albumin levels, **I** urine albumin levels, **J** albumin to creatinine ratios (ACR), and **K** blood urea nitrogen (BUN) levels of Ctrl and KO mice after PBS and LPS injection (n = 6–12 per group). **L** PAS staining of kidney sections in Ctrl and KO mice under PBS and LPS conditions; the arrow is pointing to the expansion of Bowman space in response to LPS treatment. Scale bar = 50 μM. **M** Immunofluorescence of Nephrin (green), PKM2 (red), and nuclear DNA using DAPI (blue) in kidney sections obtained from both genotypes injected with PBS or LPS. Scale bar = 50 μM. In **D–K,** *p < 0.05, **p < 0.01 indicate a significant difference between PBS- and LPS-injected mice. ^&^*p* < 0.05, ^&&^*p* < 0.01 indicate a significant difference between Ctrl and KO mice

To evaluate the possible contribution of PKM2 to the pathology of acute kidney injury (AKI), we generated mice lacking the expression of PKM2 in podocytes. Briefly, we crossed homozygous PKM2-floxed mice with podocin-cre transgenic mice, as described in the methods section. The expression of PKM2 was evaluated in total kidney lysates by immunoblotting and qRT-PCR. PKM2 protein levels were slightly lower in PKM2 KO mice compared to their WT control littermates, but the difference was not statistically different (Fig. 2A). On the other hand, isolated podocytes from PKM2 KO mice exhibited a significant reduction in PKM2 expression at protein and RNA levels (Fig. 2B) with no significant alterations in the levels of its splicing isoform; pyruvate kinase isozymes M1 (PKM1; Fig. 2C). In addition, we evaluated the expression of PKM2 in other PKM2 expressing tissues (including pancreas, lungs, spleen, and adipose) and found no differences in PKM2 expression in PKM2 KO mice compared to controls (Additional file 1: Figure S2). In line with these findings, immunostaining of PKM2 in kidney sections of WT and PKM2 KO mice revealed a significant decrease in PKM2 expression in podocytes of the latter group, further confirming PKM2 podocyte-specific deletion (Fig. 2M).

Next, we examined the effects of podocyte-specific deletion of PKM2 on LPS-induced AKI and found that, while the body weight of control mice was significantly reduced 24 h after LPS injection, podocyte PKM2 KO mice exhibited a significant reduction in weight loss compared to controls upon LPS injection (Fig. 2D). In addition, LPS treatment resulted in higher kidney weights and kidney to body weight ratio in WT mice, while PKM2 deficiency attenuated these effects (Fig. 2E–F). Moreover, an assessment of serum and urine albumin levels revealed that exposure to LPS resulted in a significant increase in urinary protein and albumin excretion in control mice paralleled with a reduction in serum albumin concentrations (Fig. 2G–I). On the other hand, mice with a specific deletion of PKM2 in podocytes exhibited a significantly less pronounced alteration in serum and urine albumin levels (Fig. 2G–I). The reduction in serum albumin levels in control mice is consistent with previous reports [31, 43] and suggests a decrease in glomerular filtration capacity and renal function in general. To validate this hypothesis, we examined changes in urine albumin to creatinine ratio (ACR) and blood urea nitrogen (BUN) before and after LPS treatment. We found that LPS caused a significant increase in urinary ACR (Fig. 2J) and BUN (Fig. 2K). These effects were more severe in the control group than podocyte PKM2 KO mice (Fig. 2J–K).

AKI is often associated with histological and structural changes in glomeruli characterized by substantial morphological alterations within the tubules. These changes may occur in response to excess proteinuria and albumin loss [44], and include brush border loss and cytoplasmic vacuolization. As a result, tubular cell fragmentation or complete detachment to the lumen, and the formation of obstructive casts may occur [45, 46]. Additionally, dilation in tubules may be observed as a result of altered intraluminal pressure and cytoskeleton arrangement [47]. Since impeded flow caused by inflammation and obstruction is common in injured tubules, fluids may leak into the denuded basal membrane leading to interstitial edema. Accordingly, we performed a periodic acid–Schiff (PAS) staining to examine the effects of PKM2 deficiency on LPS-induced alterations to the structure of the mesangial matrix and potential modifications of the glomerular basement membrane (GBM). Consistent with published studies, LPS induced significant structural and morphological alterations to the tubules and to the brush border in control mice as judged by the level of cytoplasmic fragmentation, nuclear loss, epithelial sloughing, and obstructive casts (Fig. 2L and Table 3). In direct support of our previously described biochemical data, PKM2 deficiency in podocytes mitigated the histological alterations caused by LPS and preserved glomerular structure (Fig. 2L). Notably, these findings are consistent with those from the recent study by Wu and colleagues demonstrating that shikonin-mediated inhibition of PKM2 alleviated LPS-induced kidney injury and histopathological alterations to renal tubular epithelial cells [28] emphasizing the role of PKM2 in renal injury.

**Table 3:** Histology assessment of kidneys injury in control and PKM2 KO mice. We examined the tubules of the cortical and corticomedullary areas using PAS-stained renal sections obtained from PBS or LPS-treated wild type (Ctrl) and podocyte PKM2 knockout (KO) mice and scored the degree of injury as detailed in the methods section

	Cortical area	Corticomedullary junction
Ctrl-PBS	1.36 ± 0.78	0.85 ± 0.41
KO-PBS	0.51 ± 0.23	0.22 ± 0.10
Ctrl-LPS	16.29 ± 6.59*	14.54 ± 5.78*
KO-LPS	2.77 ± 1.16*^#^	2.61 ± 1.19^#^

*p < 0.05 indicates a significant difference between PBS- and LPS-injected mice. ^#^*p* < 0.05 indicates a significant difference between Ctrl and KO mice

Nephrin is an essential protein in the slit diaphragm and plays a critical role in maintaining the glomerular filtration barrier. Loss of nephrin in the urine is a hallmark of AKI and podocyte injury in humans and rodents [48]. Thus, to confirm whether PKM2 deletion protects mice against LPS-induced AKI, we examined changes in nephrin levels upon exposure to LPS by immunofluorescence microscopy. Consistent with previous reports [40], we show that LPS treatment leads to a decrease in nephrin expression in podocytes, both in vivo and in vitro (Fig. 1). Furthermore, a significant reduction in nephrin was observed in kidney sections from LPS treated control mice (Fig. 2M). However, mice with PKM2 deficiency in podocytes retained a significantly higher level of nephrin, confirming the protective effects of PKM2 depletion against LPS-induced histological and biochemical alterations.

### PKM2 podocyte deficiency attenuates LPS induced renal inflammation and apoptosis in vivo

**Fig. 3:**
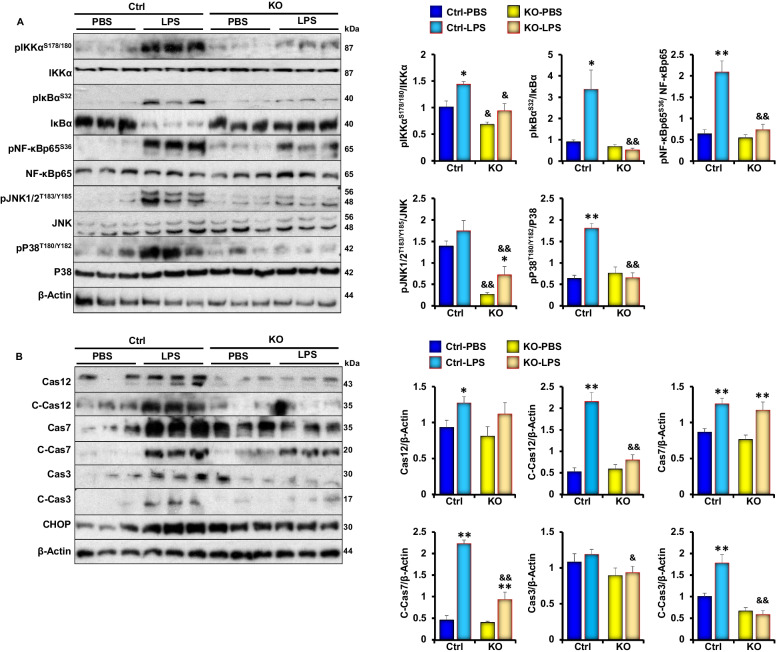
Podocyte specific deletion of PKM2 attenuates LPS-Induced inflammation and apoptosis in Vivo. **A** Representative immunoblots (left panel) and bar graph quantitative assessment (right panel) of major signal transduction molecules involved in the NF-κB (pIKKα^S178/S180^, IKKα, pIκBα^S32^, IκBα, pNF-κBp65^S36^, NF-κBp65), MAPK (pJNK1/2^T183/Y185^, JNK, pP38^T180/Y182^, P38) signaling pathways, and β-Actin as a loading control in whole kidney lysates of control (Ctrl) and podocyte PKM2 knockout mice (KO) mice harvested 24 h after PBS or LPS injection (n ≥ 6/group). *p < 0.05, **p < 0.01 indicate a significant difference between PBS- and LPS-injected mice. ^&^*p* < 0.05, ^&&^*p* < 0.01 indicate a significant difference between Ctrl and KO mice. **B** Representative immunoblots (left panel) and bar graph quantification (right panel) of apoptotic markers: Caspase 12, 7, and 3, their cleaved forms, and CHOP in whole kidney lysates of control (Ctrl) and podocyte PKM2 knockout mice (KO) mice harvested 24 h after PBS or LPS injection (n ≥ 6/group). *p < 0.05, **p < 0.01 indicate a significant difference between PBS- and LPS-injected mice. ^&^*p* < 0.05, ^&&^*p* < 0.01 indicate a significant difference between Ctrl and KO mice

Next, we sought to investigate whether PKM2 deficiency mitigates LPS-induced inflammation and apoptosis since these pathways have been identified to play a key role in mediating the pathogenesis of AKI during its early phases. Previous studies have shown that LPS treatment mediates renal injury through activating the inflammatory signaling pathways [49]. NF‐κB is a major regulator of inflammatory cytokine expression [50] and a mediator of LPS-induced renal injury [51]. Therefore, we examined the expression and phosphorylation of key proteins involved in the transduction of the NF‐κB signaling pathway in response to LPS in control and podocyte PKM2 KO mice. As shown in Fig. 3A, LPS treatment induced the phosphorylation and activation of NF‐κB, p38, and JNK in both genotypes, but this induction was significantly lower in the KO mice. Similarly, apoptotic markers (cleaved caspases 3, 7 and 12, and CHOP) were also lower in podocyte PKM2 KO mice compared to controls (Fig. 3B). While these findings demonstrate that PKM2 deficiency attenuates LPS-induced alterations in podocyte homeostasis and apoptotic cell death, they also suggest that PKM2 acts upstream of inflammation during podocytes’ response to LPS.

### PKM2 deficiency in cultured podocytes ameliorates LPS induced inflammation and apoptosis

**Fig. 4:**
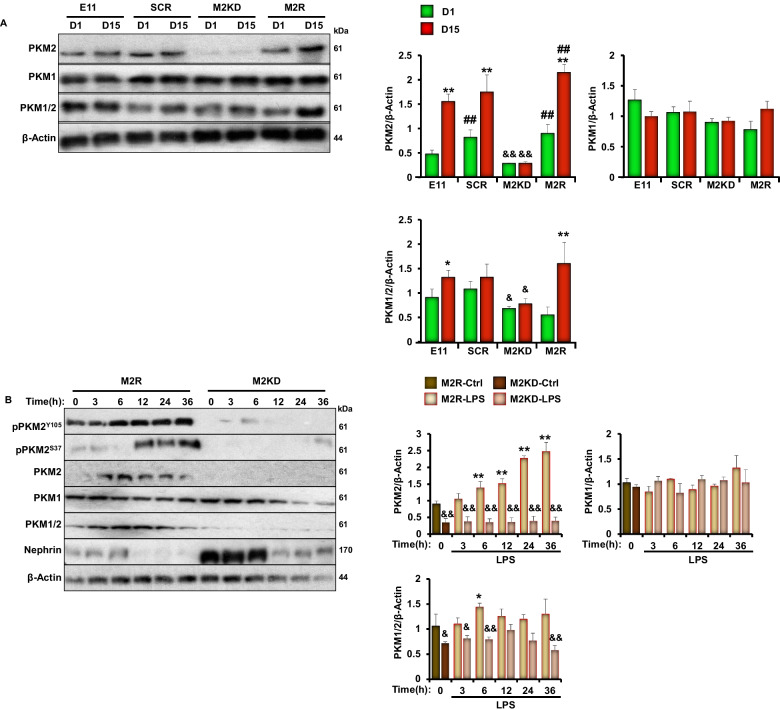
PKM2 deficiency in cultured E11 podocytes alleviates LPS-induced nephrin loss. **A** Representative immunoblots (left panel) and bar graph quantitative assessment (right panel) of PKM2, PKM1, and PKM1/2 levels in undifferentiated (day 1; D1) and differentiated (D15) E11 murine podocytes infected with lentivirus particles carrying scramble-shRNA (SCR), shRNA targeting PKM2 (M2KD) or an open reading frame of the human DNA (M2R). β-Actin was used as a loading control. *p < 0.05, **p < 0.01 indicate a significant difference between differentiated and undifferentiated cells. ^#^*p* < 0.05, ^##^*p* < 0.01 indicate a significant difference between the indicated cell and control E11 cells exposed to the same treatment. **B** Representative immunoblots (left panel) and bar graph quantitative assessment (right panel) of pPKM2^Y105^, pPKM2^S37^, PKM2, PKM1, PKM1/2, and Nephrin in differentiated M2R and M2KD podocytes treated with LPS for the indicated durations. β-Actin was used as a loading control. Data is representative of at least three independent experiments. *p < 0.05, **p < 0.01 indicate a significant difference between LPS-treated or non-treated cells. ^&^*p* < 0.05, ^&&^*p* < 0.01 indicate a significant difference between M2R and M2KD cells exposed to the same treatment

In an attempt to elucidate the role of PKM2 in podocyte function and delineate the molecular basis of the observed protective outcomes of PKM2 depletion against LPS-induced podocyte injury, we conducted in vitro studies using murine E11 cells. We also used the lentiviral shRNA approach to generate cells with stable PKM2 knockdown (M2KD), and a scramble (SCR) shRNA to generate control cells (SCR). To avoid drawing inaccurate conclusions or any experimental procedure-related off-target effects, PKM2 knockdown cells were reconstituted with a human wild type PKM2 (M2R). Since M2R cells exhibited a significant change in PKM2 levels (Fig. 4A) and a reduction in nephrin expression that was comparable to control cells generated using a scramble shRNA (SCR; Additional file 1: Figure S3), we used M2R cells as control cells in our follow-up experiments on E11 podocytes. All cells were cultured and differentiated into podocytes as previously described [30]. Immunoblot analysis shows that E11 cells infected with lentivirus carrying PKM2 shRNA exhibited a significant reduction in PKM2 levels before (Day 1; D1) and after differentiation (Day 15; D15). On the other hand, M2R cells expressed near endogenous levels of the protein (Fig. 4A). Importantly, immunoblotting for the M1 isoform did not show a significant alteration in the expression of PKM1 in PKM2 knockdown cells (M2KD). In addition, immunoblotting with an antibody that binds to both M1 and M2 isoforms showed a significant reduction in total PKM levels, further confirming the knockdown of PKM2 (Fig. 4A). Notably, while PKM2 level increases with differentiation in both M2R and SCR cells, M2KD cells maintained a significantly lower level of PKM2 throughout the differentiation process (Fig. 4A and Additional file 1: Figure S3A). Of note, the increase in PKM2 in differentiated cells is consistent with a recent study [29] and suggests a potential role for PKM2 in podocyte differentiation, thus warranting additional investigation. Additionally, we observed a slight, albeit not statistically different, reduction in PKM1 expression on Day 15 compared to Day 1 (Fig. 4A) in both E11 and M2R cells, but not in M2KD.

It is well established that podocytes are terminally differentiated cells required for supporting the integrity of the GBM [52]. The magnitude of podocyte injury strongly correlates with proteinuria and kidney damage [53, 54]. Thus, to define the role of PKM2 in podocyte injury, we examined changes in PKM2 expression and phosphorylation in response to LPS treatment for 3, 6, 12, 24, and 36 h. Western blotting analyses revealed a time-dependent increase in PKM2 expression, concomitant with a reduction in nephrin levels (Fig. 4B). PKM1 levels on the other hand, remained unchanged in both control (M2R) and M2KD cells exposed to LPS (Fig. 4B and Additional file 1: Figure S3A). Furthermore, bioenergetic profiles of M2KD and M2R podocytes revealed that upon treatment with LPS, M2R podocytes shifted toward an increase in glycolysis as judged by the increase in ECAR following addition of glucose (Additional file 1: Figure S3B–C). However, PKM2 KD podocytes failed to show this metabolic shift following LPS treatment. Together, these findings prove the regulation of PKM2 expression and glycolysis in podocytes upon LPS challenge and are in support of our in vivo findings (Figs. 1, 2).

**Fig. 5:**
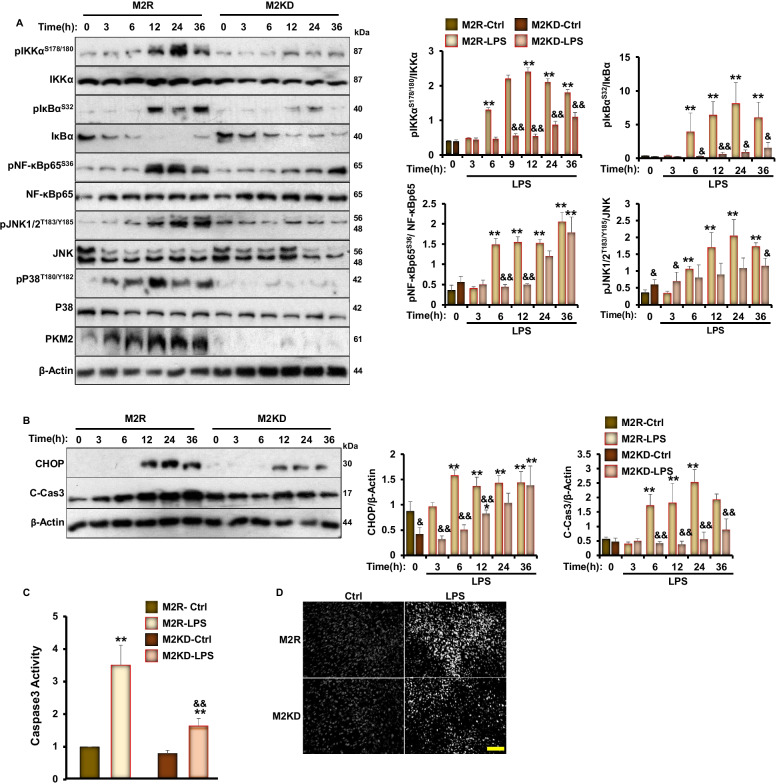
PKM2 deficiency ameliorates LPS-induced inflammation and apoptosis in cultured E11 podocytes. **A** Representative immunoblots (left panel) and bar graph quantitative assessment (right panel) of pIKKα^S178/S180^, IKKα, pIκBα^S32^, IκBα, pNF-κBp65^S36^, NF-κBp65, pJNK1/2^T183/Y185^, JNK, pP38^T180/Y182^, P38, and PKM2 in total cell lysates from differentiated M2R and M2KD podocytes treated with LPS for the indicated durations. β-Actin was used as a loading control. Data is representative of at least three independent experiments. *p < 0.05, **p < 0.01 indicate a significant difference between LPS-treated or non-treated cells. ^&^*p* < 0.05, ^&&^*p* < 0.01 indicate a significant difference between M2R and M2KD cells exposed to the same treatment. **B** Representative immunoblots (left panel) and bar graph quantitative assessment (right panel) of CHOP and cleaved-caspase 3 (C-Cas3) in total cell lysates from differentiated M2R and M2KD podocytes treated with LPS for the indicated durations. β-Actin was used as a loading control. Data is representative of at least three independent experiments. *p < 0.05, **p < 0.01 indicate a significant difference between LPS-treated or non-treated cells. ^&^*p* < 0.05, ^&&^*p* < 0.01 indicate a significant difference between M2R and M2KD cells exposed to the same treatment. **C** Caspase 3 activity in M2R and M2KD cells treated (LPS) or non-treated (Ctrl) with LPS for 24 h. **p < 0.01 indicate a significant difference between LPS-treated or non-treated cells. ^&&^*p* < 0.01 indicate a significant difference between M2R and M2KD cells exposed to the same treatment. **D** Representative images of chromatin condensation in Hoechst-stained M2R and M2KD podocytes treated with LPS for 24 h. Scale bar: 100 μm. Images are representative of at least three independent experiments

To decipher the contribution of PKM2 to podocyte injury, we examined whether PKM2 depletion in E11 podocytes would recapitulate the in vivo observations (Fig. 3) by evaluating the effects of PKM2 deficiency on LPS-induced NF‐κB signaling and apoptosis. As shown in Fig. 5, LPS induced the phosphorylation of IKKα, IκBα, and NF‐κB p65 in both control and M2KD cells. However, the level of activation of NF‐κB in M2KD cells was significantly reduced compared to control M2R podocytes (Fig. 5A). Similarly, LPS induced the phosphorylation of JNK in both cell lines, but to a lower extent in the M2KD cells (Fig. 5A). Moreover, PKM2 deficiency alleviated LPS-induced caspase 3 cleavage and activation (Fig. 5B, C).

Activation of caspase 3 leads to the disruption of the DNA repair machinery and results in chromatin condensation and nuclear fragmentation [55]. Thus, we performed Hoechst staining and examined changes in the number of Hoechst positive cells in response to LPS treatment in both M2R and M2KD podocytes. Exposure to LPS for 24 h resulted in a significantly higher chromatin condensation level, which is indicative of apoptotic cell death in both cell lines. However, M2KD podocytes exhibited a reduced number of apoptotic cells compared to M2R cells (Fig. 5D). Collectively, these findings are in line with the in vivo protective outcomes of PKM2 depletion against LPS-induced podocyte injury.

### Inhibition of beta-catenin mediates the beneficial effects of PKM2 deficiency against LPS-induced podocyte injury

**Fig. 6:**
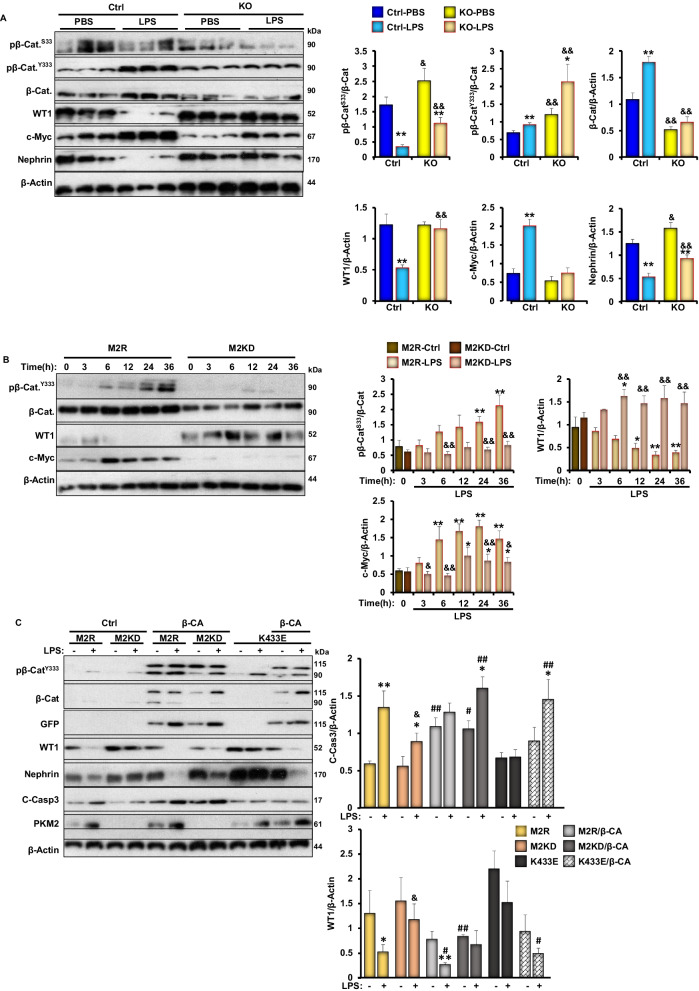

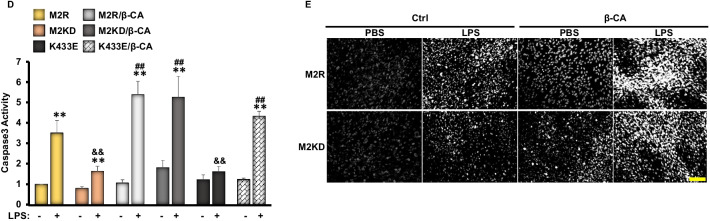
PKM2 depletion suppress LPS-induced-β-catenin activation in kidneys and E11 cultured podocytes. **A** Representative immunoblots (left panel) and bar graph quantitative assessment (right panel) of pβ-Catenin^S33^, pβ-Catenin^Y333^, β-Catenin and its downstream targets (WT1 and c-Myc), and nephrin in total kidney lysates harvested from C57bl6/J wild type mice 24 h after PBS or LPS injection (n ≥ 6/group). β-Actin was used as a loading control. *p < 0.05, **p < 0.01 indicate a significant difference between PBS- and LPS-injected mice. ^&^*p* < 0.05, ^&&^*p* < 0.01 indicate a significant difference between Ctrl and KO mice. **B** Representative immunoblots (left panel) and bar graph quantitative assessment (right panel) of pβ-Catenin^Y33^, β-Catenin, WT1, and c-Myc in total cell lysates from differentiated M2R and M2KD podocytes treated with LPS for the indicated durations. β-Actin was used as a loading control. Data is representative of at least three independent experiments. *p < 0.05, **p < 0.01 indicate a significant difference between LPS-treated or non-treated cells. ^&^*p* < 0.05, ^&&^*p* < 0.01 indicate a significant difference between M2R and M2KD cells exposed to the same treatment. **C** Representative immunoblots (left panel) and bar graph quantitative assessment (right panel) of pβ-Catenin^Y333^, β-Catenin, GFP, WT1, nephrin, C-Caspase 3, and PKM2 in in total cell lysates from LPS treated or non-treated M2R, M2KD transfected with empty pCS2 + vector (Ctrl), pCS2 plasmid expressing the constitutively active β-catenin mutant (β-CA), or the pLHCX vector expressing the K433E mutant of PKM2. β-Actin was used as a loading control. Data is representative of at least three independent experiments. *p < 0.05, **p < 0.01 indicate a significant difference between LPS-treated or non-treated cells. ^&^*p* < 0.05, ^&&^*p* < 0.01 indicate a significant difference between M2R and M2KD cells exposed to the same treatment. ^#^*p* < 0.05, ^##^*p* < 0.01 indicate a significant difference between the indicated cell overexpressing the constitutively active mutant of β-catenin (β-CA) or the K433E mutant of PKM2 and M2KD cells exposed to the same treatment. **D** Caspase 3 activity in M2R, M2KD cells in total cell lysates from LPS treated or non-treated M2R, M2KD transfected with empty pCS2 + vector (Ctrl), pCS2 plasmid expressing the constitutively active β-catenin mutant (β-CA) plasmids, or the pLHCX vector expressing the K433E mutant of PKM2. Data is representative of at least three independent experiments. *p < 0.05, **p < 0.01 indicate a significant difference between LPS-treated or non-treated cells. ^&^*p* < 0.05, ^&&^*p* < 0.01 indicate a significant difference between M2R and M2KD cells exposed to the same treatment. ^#^*p* < 0.05, ^##^*p* < 0.01 indicate a significant difference between the indicated cell overexpressing the constitutively active mutant of β-catenin (β-CA) or the K433E mutant of PKM2 and M2KD cells exposed to the same treatment. **E** Representative images of chromatin condensation in Hoechst-stained M2R and M2KD podocytes transfected with empty pCS2 + vector (Ctrl) or a pCS2 plasmid expressing the constitutively active β-catenin mutant (β-CA). Scale bar: 100 μm. Images are representative of at least three independent experiments

A growing body of evidence implies that Wnt-β catenin is critical for podocyte differentiation and homeostasis [56]. As previously demonstrated, pharmaceutical activation of β-catenin induced proteinuria in mice, while specific deletion of β-catenin in podocytes ameliorated adriamycin-induced proteinuria [57]. Given that nuclear PKM2 has been demonstrated to induce β-catenin transactivation in human cancer cells [58], we sought to explore both, the in vivo and in vitro podocyte specific effects of PKM2 deficiency on β-catenin expression and activity. In agreement with previous reports [40], exposure to LPS induced the activation of β-catenin both in mice and in E11 podocytes, as judged by the higher total protein levels of β-catenin and the increase in its phosphorylation on tyrosine 333 (Y333; Fig. 6A, B). Furthermore, previous studies using human glioblastoma cells have shown that c-Src-mediated phosphorylation of β-catenin at Y333 prevents its degradation and promotes its activation and localization to the nucleus in order to regulate the expression of numerous genes including c-Myc [58]. Consistent with these findings, our data show increased c-Myc expression in response to LPS in M2R cells. However, PKM2 deficiency, both in mice and in E11 podocytes, alleviated LPS-induced changes in β-catenin expression and its Y333 phosphorylation, and mitigated LPS-induced expression of c-Myc (Fig. 6A, B). It is worth mentioning that phosphorylation of β-catenin on one of the three glycogen synthase kinase-3 (GSK-3) target sites [serine 33 (S33) and 37 (S37), and threonine 41 (T41)] has been previously demonstrated to promote the polyubiquitination of β-catenin on lysine 19 (K19) and its subsequent proteasomal degradation [59]. Here, we demonstrate a significant decrease in β-catenin S33 phosphorylation in WT mice in response to LPS (Fig. 6A), further confirming the activation of β-catenin in response to LPS. Mice with PKM2 deletion in podocytes; however, retained a higher S33 phosphorylation compared with controls. Similar results were obtained using E11 podocytes (Fig. 6B).

Previous studies have shown that β-catenin antagonizes the function of Wilms’ Tumor 1 (WT1) [60], a critical transcription factor essential for maintaining healthy podocyte integrity [61]. Thus, we sought to examine the effect of PKM2 deletion on WT1 expression. Accordingly, we evaluated its expression in total kidney lysates under PBS or LPS conditions by Western blotting. Exposure to LPS induced a significant loss in WT1 in control mice, concomitant with a significant decrease in nephrin levels (Fig. 6A). Although similar effects were seen in mice lacking PKM2 in podocytes, the extent of WT1 loss was significantly reduced (Fig. 6A). Similarly, LPS induced a time-dependent reduction in WT1 expression in both M2R and M2KD cells, but the reduction was significantly lower in the latter (Fig. 6B). These observations highlight the role of β-catenin in mediating PKM2’s function in podocytes under stress conditions. To further validate this hypothesis, we transfected podocytes with a constitutively active mutant of β-catenin (β-CA), which resulted in a significant reduction in nephrin and WT1 protein levels (Fig. 6C), concomitant with increased cleavage and subsequent activation of caspase 3 (Fig. 6C, D) in M2R cells. Notably, β-catenin overexpression also reversed the beneficial effects of PKM2 deficiency against LPS-induced cell death in cultured podocytes (Fig. 6E). Last, since recent studies have shown that the PKM2 lysine 433 (K433) residue mediates PKM2 binding to β-catenin upon its phosphorylation on Y333 by c-Src through its lysine 433 residue [62], we sought to investigate the significance of this physical interaction in the context of LPS-induced podocyte injury. Accordingly, we overexpressed a human PKM2 mutant lacking the ability to bind to tyrosine-phosphorylated peptides (K433E) [42, 58] in M2KD cells along with the constitutively active mutant of β-catenin. As shown in Fig. 6C, D, the overexpression of PKM2-K433E mutant mirrored PKM2 deficiency and prevented LPS-induced reduction in WT1 levels. However, M2KD cells overexpressing both constitutively active forms of β-catenin and PKM2 (β-CA/K433E) exhibited a significant reduction in WT1 levels, and a significant increase in caspase 3 cleavage, suggesting that the physical interaction between PKM2 and β-catenin is necessary for the detrimental role of both proteins to podocytes homeostasis.

## Discussion

Acute kidney injury is a common consequence of sepsis that is becoming increasingly prevalent both globally and in the US. The glycolytic enzyme PKM2 has emerged as a key player in the metabolic remodeling that occurs during acute exposure to endotoxins and the resultant inflammatory cascade that impairs organ function and underlies sepsis-induced mortality. Inhibition of PKM2 in immune cells and in the lung has been shown to prevent LPS-induced inflammation, tissue damage, and mortality. Additionally, emerging evidence has linked PKM2 in the kidneys to renal function and proteinuric diseases. In a study by Kim and colleagues using an experimental model of cisplatin-induced AKI in rats, PKM2 expression was significantly increased in the renal medulla and cortex after cisplatin treatment [63]. Consistent with these findings, cisplatin treatment of human kidney (HK-2) cells increased anaerobic glycolysis and promoted a metabolic shift from pyruvate to lactate to produce energy [64]. While there have been efforts to characterize the role of PKM2 in renal function, particularly under conditions of hyperglycemia-induced glomerular injury, its contribution to the pathophysiology of AKI remains largely unexplored.

Investigations targeting the metabolic states of renal cells in health and diseases continue to uncover novel functions of this enzyme, PKM2, within the juxtaglomerular apparatus in the renal cortex. Mice with podocyte-specific deletion of PKM2 exhibited enhanced hyperglycemia-induced albuminuria and glomerular injury. Conversely, pharmacological activation of PKM2 using TEPP-46, a PKM2 selective activator that promotes PKM2 tetramerization while blocking its PKM2 nuclear translocation, ameliorated hyperglycemia-induced mitochondrial dysfunction and kidney injury in diabetic Nos3‐/‐ and DBA/2J mice. Furthermore, cultured mouse podocytes treated with TEPP-46 exhibited a significant reduction in mitochondrial dysfunction and cell death caused by prolonged exposure to high glucose concentrations [65]. Pharmacological activators of PKM2 were also shown to protect murine podocytes from accumulation of sorbitol and other cytotoxic glucose metabolites that are induced by chronic hyperglycemia in mouse podocytes [65]. While these studies identify PKM2 as a significant contributor to the pathogenesis of diabetic nephropathy and support the therapeutic potential of pharmacological activators of PKM2 in preventing kidney injury, other studies demonstrated a potential nephroprotective effect of PKM2 pharmacological inhibition. Notably, PKM2 inhibition using shikonin in rat renal tubular epithelial cells alleviated hyperglycemia-induced alterations in mitochondrial membrane potential and resulted in a significant increase in oxidative stress. Additionally, treatment with shikonin reduced the level of apoptosis caused by high glucose levels in these cells through upregulating the antioxidant defense system [66] and inhibition of apoptosis [28]. These findings are in agreement with other studies demonstrating the nephroprotective effects of PKM2 inhibition against septic acute kidney injury. Indeed, in a study by Kawara and colleagues, the pre-treatment with shikonin protected mice from LPS-induced oxidative stress in the kidneys, reduced the levels of circulating pro-inflammatory cytokines [Interleukin 6 (IL-6) and TNF-α], and prevented kidney injury and alterations in renal function [67]. In another study, shikonin-mediated pharmacological inhibition of PKM2 alleviated LPS-induced endotoxemia and sepsis, and improved survival [22]. Likewise, administration of shikonin decreased LPS-induced secretion and release of the pro-inflammatory mediators TNFα, IL-1β, and high mobility group box 1 (HMGB1). Shikonin also prevented the activation of the NLRP3/caspase-1/IL-1β inflammasome pathway and increased the survival rate of Balb/c mice with LPS-induced lethal endotoxemia and caecal ligation and puncture–induced sepsis [22]. Collectively, these studies suggest that pharmacologic inhibition of PKM2 may provide a new therapeutic approach to treat septic AKI.

In sepsis-induced AKI, podocytes are a primary target of circulating endotoxin because of their anatomical location in the glomerulus. Structural damage to podocytes can result in sustained proteinuria that links AKI to chronic renal disease. Although PKM2 is the primary pyruvate kinase isoform in mature podocytes, its role in sepsis-induced AKI has not been explored. Based on the aforementioned evidence, we speculated that PKM2 deletion in podocytes might also yield nephroprotective effects and alleviate kidney injury in the contest of LPS-induced endotoxemia and sepsis. Furthermore, because anaerobic respiration is the main source of energy in podocytes [68] under normal physiological conditions, LPS treatment may cause podocytes to undergo a metabolic switch from the anaerobic pathway to aerobic glycolysis, resulting in increased toxicity and cell death. Thereby, PKM2 deletion may prevent this shift and protect podocytes from the harmful effects of LPS. Although these mechanisms are yet to be confirmed in podocytes, prior studies have shown that the induction of aerobic glycolysis in tubular epithelial cells during the course of acute kidney injury caused by ischemic reperfusion was concomitant with increased lactate production, mitochondrial dysfunction and reduced mitochondrial mass, tubular atrophy, and renal fibrosis [69]. Recently, Ran and colleagues provided evidence for the role of the Warburg effect in promoting mitochondrial dysfunction and kidney injury in a mouse model of caecal ligation and puncture-induced AKI and in normal human kidney cells treated with LPS [70]. Furthermore, in a retrospective cohort of critically ill patients with AKI, impaired renal glucose metabolism was suggested to be a key determinant factor of mortality associated with AKI. Supplementation with thiamine, a key factor in aerobic glycolysis, increased lactate clearance and correlated with better health outcomes in humans [71]. Notably, renal PKM2 expression and lactate levels were shown to be upregulated in renal biopsies and urine of patients diagnosed with AKI. As such, PKM2 and its glycolytic function were suggested as novel independent early detection biomarkers for AKI [64, 72] emphasizing the importance of targeting the glycolytic function of PKM2 for the treatment of AKI. This hypothesis is supported by other studies investigating the role of aerobic glycolysis in sepsis-induced organ dysfunction. In these studies, inhibition of glycolysis protected against LPS-induced injury of the lungs [73] and heart [74] and promoted the migration of neutrophils to the infected sites [75]. Nonetheless, we must also acknowledge the potential contribution of the non-glycolytic functions of PKM2 to the pathogenesis of AKI. Indeed, PKM2 can also translocate to the nucleus and modulate expression of signaling pathways, independent of its role as a glycolytic enzyme. This non-glycolytic role of PKM2 was demonstrated to promote inflammation and contributes to the etiology of various inflammatory diseases including rheumatoid arthritis [76], inflammatory bowel disease [77], atherosclerotic coronary artery diseases [27], and sepsis [12]. Genetic ablation of PKM2 in myeloid cells exerts a protecting effect against LPS induced endotoxemia in mice, while the pharmaceutical inhibition of PKM2 using shikonin improved mice survival rate [23]. Since AKI is an inflammatory disease associated with podocyte injury, it is reasonable to assume that PKM2 in podocytes contributes to sepsis-induced AKI. Thus, we examined the effects of PKM2 deficiency to AKI-associated inflammation in an experimental model of LPS-induced podocyte injury.

In the present study, we report that LPS treatment increased the expression of PKM2 and increased its phosphorylation in podocytes. These effects were concomitant with proteinuria and kidney injury. Notably, podocyte specific PKM2 deletion ameliorated LPS induced proteinuria and preserved serum albumin levels. Furthermore, PKM2 depletion was associated with reduced structural and morphological alterations in response to LPS. These protective effects were concomitant with reduced activation of major pathways that have been demonstrated to mediate the etiology of AKI. Indeed, PKM2 deficiency in podocytes attenuated LPS-induced inflammation and apoptosis in vivo and ex vivo. On the other hand, PKM2 depletion prevented LPS-induced loss of nephrin, an essential component in the development and maintenance of podocyte slit diaphragm. Mechanistically, the deletion of PKM2 reduced the activation of β-catenin and its downstream target c-Myc but preserved the levels of Wilms' tumor-1 (WT1), a contributing factor to the maintenance of podocyte homeostasis. Collectively, our study demonstrates that PKM2 is a critical player in LPS-induced AKI, and targeting PKM2 might be of therapeutic value to halt the progression of AKI.

The Wnt/β-catenin signaling is a critical pathway required for various cellular processes including proliferation, differentiation, inflammation, endoplasmic reticulum (ER) stress, apoptosis, and autophagy [78, 79]. For example, activation of β-catenin by the Wnt signal has been reported to antagonize the autophagic flux in several cell models, including neuronal and colorectal carcinoma cells [80, 81]. However, in the absence of Wnt ligands, β-catenin is destined for proteasomal degradation through the coordinated action of casein kinase 1 (CK1) and the APC/Axin/GSK-3β-complex. Indeed, Wnt ligands disrupt the APC/Axin/GSK-3β-complex and promote β-catenin stabilization and the subsequent nuclear localization where it exhibits transcriptional activity [82]. Interestingly, β-catenin cytoplasmic accumulation was shown to promote its proteolytic degradation, concomitant with the induction of autophagy, and suppression of apoptosis in BCR-ABL1^+^ leukemic cells [83]. In addition, β-catenin has been shown to induce ER stress in hepatocytes [84] and endothelial cells [85], and its activation and stabilization were shown to positively correlate with increased apoptosis in several cell lines [86]. The molecular mechanisms mediating β-catenin pro-apoptotic effects are not fully characterized and involve a plethora of signaling pathways, including inflammation, ER stress, and autophagy. Interestingly, recent studies also reported that both inflammation [87] and ER stress [88] could negatively regulate the activity of β-catenin, suggesting a complex interplay amongst these three pathways that warrants additional investigation.

A growing body of literature implicates β-catenin in renal diseases. Recently, β-catenin activation and its subsequent translocation to the nucleus were demonstrated to mediate increased fluid flow shear stress (FFSS)-induced podocyte injury in solitary kidney [89]. Likewise, β-catenin activation promotes renal inflammation after uninephrectomy surgery and BSA administration [90], while its inhibition reduces apoptosis in renal mesangial cells [91]. The Wnt signaling pathway plays a pivotal role in the activation and stabilization of β-catenin in multiple proteinuric disease models including diabetic neuropathy [92] and LPS induced AKI [40]. Prolonged activation of β-catenin signaling promotes the progression of AKI to chronic kidney disease (CKD) and the onset of renal fibrosis [93]. Furthermore, renal biopsy obtained from nephrotic syndrome patients with either small focal lesion or acute tubular necrosis revealed an increase in kidney injury score and Wnt/β-catenin expression in the latter [94]. Moreover, genetic deletion of β-catenin in podocytes was protective against adriamycin-induced renal injury, while the activation of β-catenin mediated by overexpressing Wnt1 exacerbated albuminuria and loss of nephrin [57]. However, transient activation of β-catenin was suggested to promote renal repair after injury, indicating a dual role for β-catenin in kidney diseases [95]. More importantly, siRNA mediated silencing of β-catenin suppressed apoptosis in renal mesangial cells after TNF-α treatment [91]. Likewise, inhibition of Wnt/β-catenin pathway mitigated LPS-induced renal injury and resulted in less structural and functional defects [40]. These studies highlight the importance of targeting the Wnt/β-catenin signaling pathway and its regulators for the treatment of renal diseases and the prevention of podocyte depletion.

In a recent study, PKM2 was reported to regulate the transactivation of β-catenin in response to epidermal growth factor receptor (EGFR) activation [58] and was also suggested to play a critical role in mediating β-catenin nuclear localization in renal cell carcinoma treated with metformin. On the other hand, silencing PKM2 reduced the nuclear accumulation of β-catenin [96]. Likewise, the downregulation of PKM2 expression in Hep3B cells suppressed β-catenin activity and promoted its proteolytic degradation [97]. In line with these reports, our study shows that PKM2 depletion protected against the induction of apoptosis in mice and cultured podocytes. We also demonstrate that the beneficial effects of PKM2 deletion against LPS-induced apoptosis were mediated, at least in part, through the suppression of β-catenin. LPS treatment induced the phosphorylation of β-catenin at Y333, a site required for PKM2 binding [58]. The disruption of PKM2/β-catenin interaction ameliorated LPS induced apoptosis and recapitulated the beneficial effects of PKM2 depletion against LPS-induced apoptosis in cultured podocytes. Additionally, our study suggests that PKM2 is an upstream regulator of β-catenin and provides novel insights into the molecular contribution of PKM2 in podocytes and its role in regulating β-catenin expression levels and activation in response to LPS. We demonstrate that the increased PKM2 expression in response to LPS was concomitant with increased levels of β-catenin and its Y333 phosphorylation both in vitro and in vivo. Whereas PKM2 deletion suppressed LPS induced β-catenin and Y333 phosphorylation and promoted β-catenin S33 phosphorylation in total kidney lysates and cultured podocytes. Based on previous studies, the phosphorylation of β-catenin at Y333 promotes the activity and localization of β-catenin to the nucleus [98]. In contrast, S33 phosphorylation reduces β-catenin activity and promotes its ubiquitination [99]. Since β-catenin has been reported to promote the expression of c-Myc [79] while antagonizing the expression and function of WT1 [60], we further confirmed the regulatory effect of PKM2 on β-catenin by examining c-Myc and WT1 expression. In the current study, we show that the reduced expression of β-catenin in PKM2 deficient podocytes was concomitant with reduced c-Myc and elevated WT1 levels. We also demonstrate that stabilizing β-catenin abolishes the effects of PKM2 deletion on c-Myc, WT1, and nephrin expression, as well as caspase 3 activation and the induction of cell death. These data suggest that β-catenin mediates the beneficial effects of PKM2 deficiency on podocytes homeostasis and function, arguing for a novel role of PKM2 in AKI.

## Conclusions

In summary, our study depicts a novel mechanism through which PKM2 plays a critical role in the pathophysiology of AKI and demonstrates that targeting PKM2 may be a viable option for the reversal of podocyte damage and the progressive loss of nephrons. Future studies to elucidate the molecular mechanisms mediating PKM2 function in other structural components of the kidney and to address the controversial contributions of PKM2 to other models of podocyte injury will be of benefit to our understanding of the complex functions of this unique enzyme in podocyte homeostasis and renal diseases.

## Supplementary Information

**Additional file 1: Figure S1. A**) mRNA levels of *Pkm2* in total kidney (n = 6/group) and primary podocytes (n = 4/group) from C57BL6 mice treated with PBS or LPS for 24 h. **B-C** Extracellular acidification (**B**) and lactate production (**C**) in differentiated E11 podocytes treated with PBS (Controls) or LPS for 6 h. In **A** and **C**, *p < 0.05, **p < 0.01 indicate a significant difference between LPS-treated or non-treated mice (**A**) or E11 podocytes (**C**). **Figure S2**. Specificity of PKM2 Deficiency in Podocytes: Representative immunoblots of PKM2, PKM1, and PKM1/2 in adipose, spleen, pancreas, and lungs of WT and KO mice (n = 6 per group). β-Actin was used as a loading control. **Figure S3**. Changes in PKM2 and Nephrin Expression in Response to LPS Treatment in M2R, SCR, and Original E11 Cells. Representative immunoblots of PKM2, PKM1, PKM1/2, and nephrin in undifferentiated (day 1; D1) and differentiated (D15) E11 murine podocytes infected with lentivirus particles carrying scramble-shRNA (SCR), shRNA targeting PKM2 (M2KD) or an open reading frame of the human DNA (M2R). When indicated, cells were treated with LPS for 24 h. β-Actin was used as a loading control. **B-C** Extracellular acidification (ECAR; **B**) and lactate production (**C**) in differentiated M2R and M2KD podocytes treated with LPS for 6 h. *p < 0.05 indicates a significant difference between LPS-treated or non-treated cells. ^&&^p < 0.01 indicates a significant difference between M2R and M2KD podocytes treated with LPS.

## Data Availability

All data generated or analysed during this study are included in this published article.

## Abbreviations

ACR: Albumin to creatinine ratio
AKI: Acute kidney injury
APC: Adenomatous polyposis coli
BUN: Blood urea nitrogen
CHOP: C/EBP homologous protein
ER: Endoplasmic reticulum
GSK: Glycogen synthase kinase
HIF 1α: Hypoxia-inducible factor 1-alpha
IL‐1β: Interleukin‐1β
IL‐6: Interleukin‐6
JNKs: C-Jun N-terminal kinases
LPS: Lipopolysaccharides
MAPK: Mitogen-activated kinases
NF-κB: Nuclear factor kappa-light-chain-enhancer of activated B cells
OXPHOS: Oxidative phosphorylation
PAS: Periodic acid–Schiff
PBS: Phosphate buffered saline
PK: Pyruvate kinase
PKM1/2: Pyruvate kinase muscle type isoform ½
TLR: Toll-like receptor
TNFα: Tumor necrosis factor‐α
WT1: Wilms’ tumor 1
XBP1: X-Box binding protein 1
