# Supplementary figures and images for: Podocyte specific deletion of PKM2 ameliorates LPS-induced podocyte injury through beta-catenin
Source: Cell Commun Signal. 2022 May 30;20:76. doi: 10.1186/s12964-022-00884-6 (PMC9150347; doi:10.1186/s12964-022-00884-6)

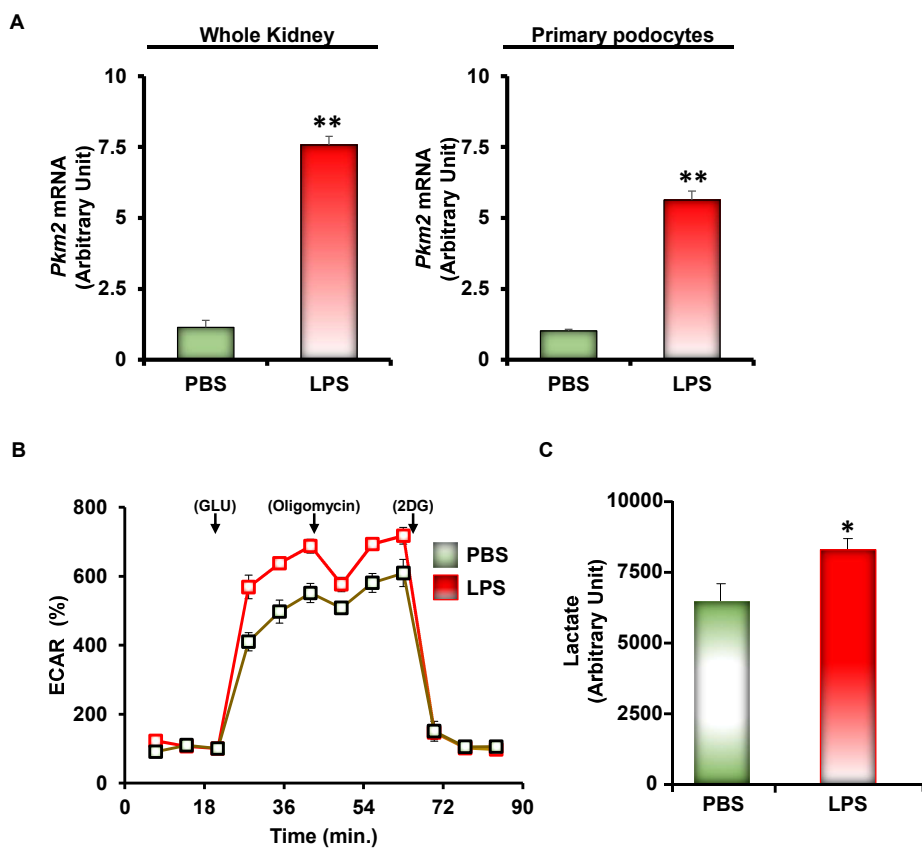

Figure S1

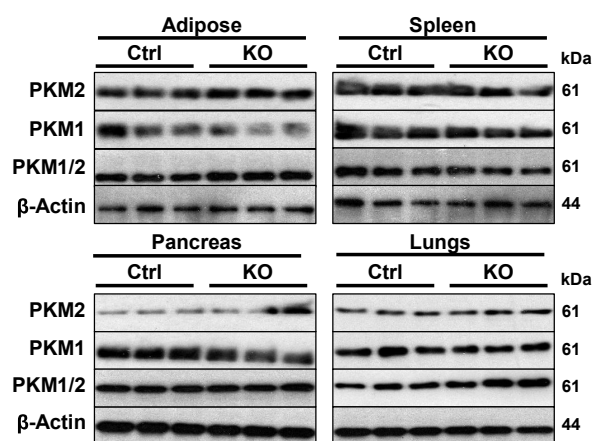

Figure S2

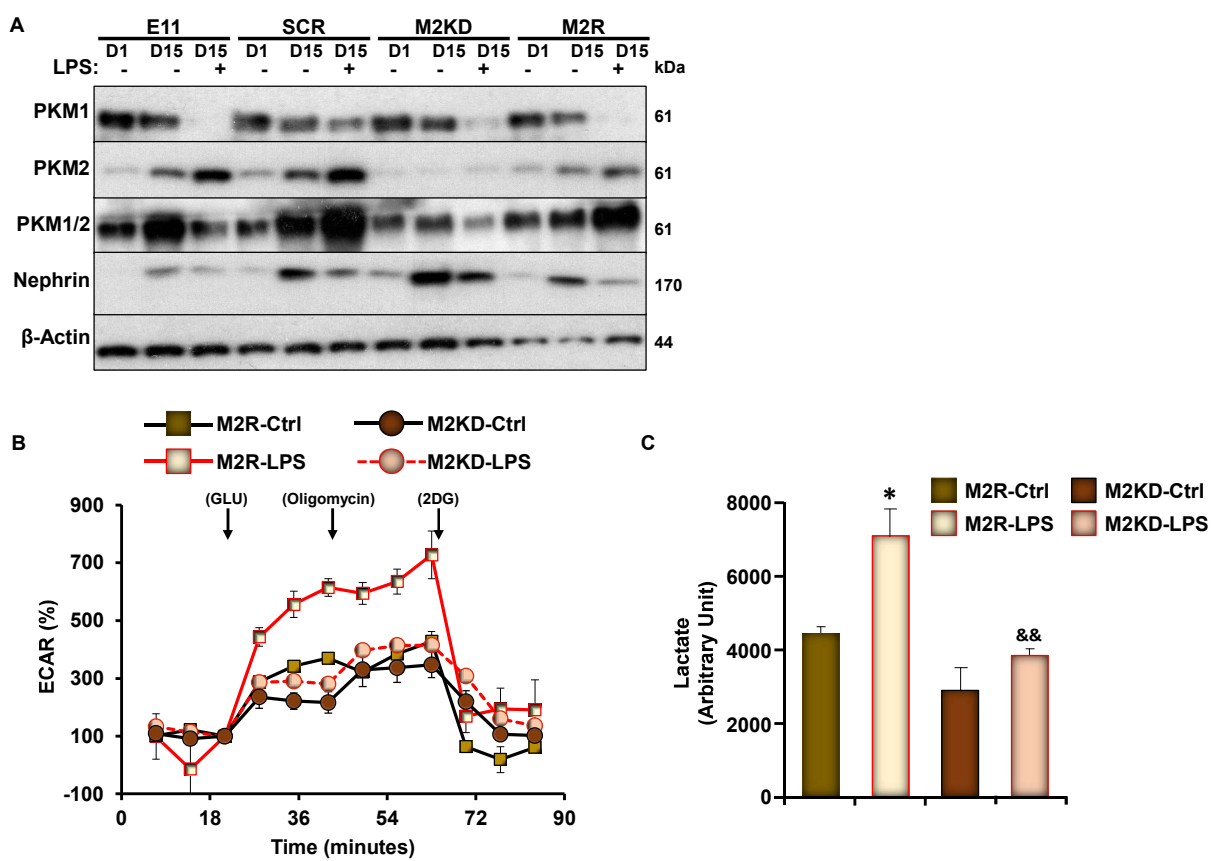

Figure S3

Supplement: Supplementary file 2 — Additional file 1: Figure S1. A) mRNA levels of Pkm2 in total kidney (n = 6/group) and primary podocytes (n = 4/group) from C57BL6 mice treated with PBS or LPS for 24 h. B-C Extracellular acidification (B) and lactate production (C) in differentiated E11 podocytes treated with PBS (Controls) or LPS for 6 h. In A and C, *p < 0.05, **p < 0.01 indicate a significant difference between LPS-treated or non-treated mice (A) or E11 podocytes (C). Figure S2. Specificity of PKM2 Deficiency in Podocytes: Representative immunoblots of PKM2, PKM1, and PKM1/2 in adipose, spleen, pancreas, and lungs of WT and KO mice (n = 6 per group). β-Actin was used as a loading control. Figure S3. Changes in PKM2 and Nephrin Expression in Response to LPS Treatment in M2R, SCR, and Original E11 Cells. Representative immunoblots of PKM2, PKM1, PKM1/2, and nephrin in undifferentiated (day 1; D1) and differentiated (D15) E11 murine podocytes infected with lentivirus particles carrying scramble-shRNA (SCR), shRNA targeting PKM2 (M2KD) or an open reading frame of the human DNA (M2R). When indicated, cells were treated with LPS for 24 h. β-Actin was used as a loading control. B-C Extracellular acidification (ECAR; B) and lactate production (C) in differentiated M2R and M2KD podocytes treated with LPS for 6 h. *p < 0.05 indicates a significant difference between LPS-treated or non-treated cells. &&p < 0.01 indicates a significant difference between M2R and M2KD podocytes treated with LPS. [file 12964_2022_884_MOESM2_ESM.pdf]
